# Effective conservative management of late-onset post-endoscopic retrograde cholangiopancreatography pancreatitis due to biodegradable stent fragments retention

**DOI:** 10.1055/a-2512-5346

**Published:** 2025-01-28

**Authors:** Irma Valeria Albergati, Andrea Anderloni, Pietro Fusaroli, Graziella Masciangelo, Fabio Monica, Andrea Lisotti

**Affiliations:** 1 Gastroenterology and Endoscopy Unit, Università degli Studi di Trieste, Cattinara Hospital, Trieste, Italy; 2 18631 Gastroenterology and Endoscopy Unit, Fondazione IRCCS Policlinico San Matteo, Pavia, Italy; 3 Gastroenterology Unit, University of Bologna, Hospital of Imola, Imola, Italy; 4 Gastroenterology and Endoscopy Unit, Cattinara Hospital, Trieste, Italy

In the last decade, biodegradable stents have been proposed to spare subsequent endoscopic retrograde cholangiopancreatography (ERCP) procedures required for stent removal 1 2 . Main adverse events in this field were migration and post-ERCP pancreatitis (PEP) 3 4 ; anecdotally, failure of stent degradation has been reported 5 .

We present the case of a 56-year-old man ( Video 1 ) who underwent an endoscopic papillectomy (EP) of a 12-mm low-grade adenoma. The patient underwent en bloc EP with a hot snare; both a prophylactic nonsteroidal anti-inflammatory drug suppository and lactated Ringer’s hydration were administered to reduce the risk of PEP. The common bile duct and pancreatic duct (PD) were non-dilatated; both biliary and pancreatic sphincterotomy were performed and an 8.5-Fr biliary plastic stent (Advantix; Boston Scientific, Marlborough, Massachusetts, USA) and a biodegradable pancreatic stent (Archimedes “Fast”) 2 mm in diameter and 6 mm long were placed ( Fig. 1 ). The Archimedes Fast degrading pancreatic stent has a declared minimal strength retention of 12 days. The procedure was uneventful and the patient was discharged after two days.

The video shows the retention of a biodegradable pancreatic stent (Archimedes “Fast”) fragment three weeks after endoscopic papillectomy, causing acute pancreatitis. Conservative management was the right choice for the patient.Video 1

**Fig. 1:**
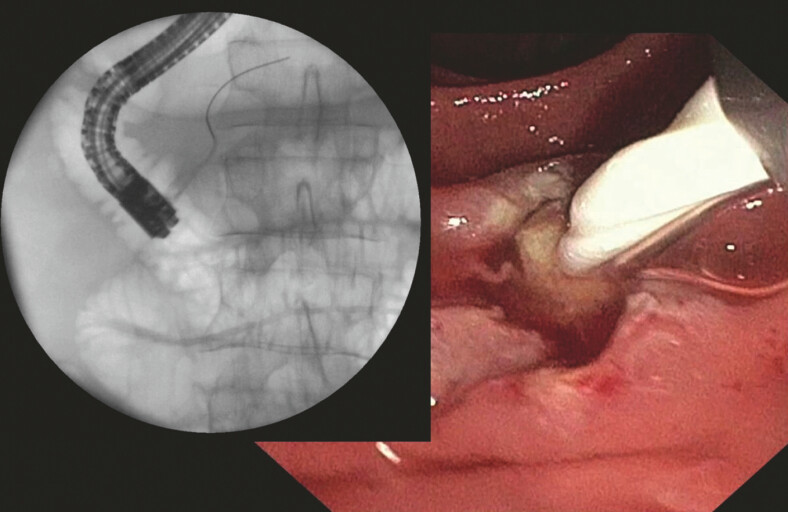
Cholangiographic and endoscopic image showing Archimedes stent insertion in the pancreatic duct (PD).

Three weeks later, the patient was admitted for mild acute pancreatitis. Computed tomography ( Fig. 2 ) showed a 6-mm retained fragment of the pancreatic stent at the level of the neck with mild upstream dilatation of the PD and peripancreatic edema. The patient received hydration and opioids. We observed prompt symptom resolution that allowed a conservative management. A magnetic resonance study one week later showed slow degradation of the retained fragment. We finally removed the biliary stent after three months, withdrawing all medical treatment. Subsequent follow-up was unremarkable.

**Fig. 2:**
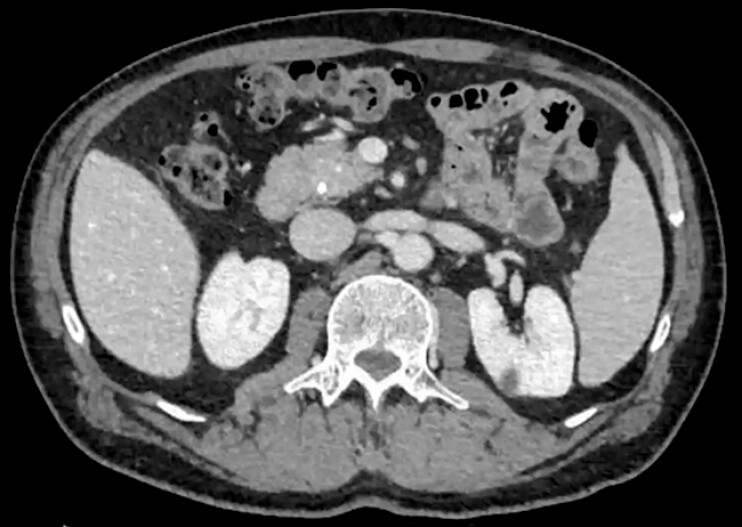
Abdominal computed tomography showing two retained stent fragments in the PD and mild PD dilatation.

Incomplete biodegradable stent dissolution should be taken into consideration among possible causes of late-onset PEP; however, conservative management could be considered a viable option along with medical and supportive treatment.

Endoscopy_UCTN_Code_TTT_1AR_2AI

## Notes

Endoscopy E-Videos https://eref.thieme.de/e-videos
*E-Videos* is an open access online section of the journal *Endoscopy* , reporting on interesting cases and new techniques in gastroenterological endoscopy. All papers include a high-quality video and are published with a Creative Commons CC-BY license. Endoscopy E-Videos qualify for HINARI discounts and waivers and eligibility is automatically checked during the submission process. We grant 100% waivers to articles whose corresponding authors are based in Group A countries and 50% waivers to those who are based in Group B countries as classified by Research4Life (see: https://www.research4life.org/access/eligibility/ ). This section has its own submission website at https://mc.manuscriptcentral.com/e-videos .
